# Mass balance study of [^14^C]Netanasvir Phosphate in healthy Chinese participants

**DOI:** 10.1128/aac.01655-25

**Published:** 2026-04-20

**Authors:** Chun-yang Zhao, Gang-yi Liu, Lin Qian, Yu-lei Zhuang, Lin Luo, Ying-jun Zhang, Hong-ming Xie, Jing Zhu, Ming Li, Qian Chen, Yun Liu, Jing-ying Jia, Yan-mei Liu

**Affiliations:** 1 Drug Clinical Trial Center, Shanghai Xuhui Central Hospital/Xuhui Hospital, Fudan University 12478, Shanghai, China; 2 Shanghai Engineering Research Center of Phase I Clinical Research and Quality Consistency Evaluation for Drugs, Shanghai, China; 3 Sunshine Lake Pharma Co., Ltd., Guangdong, China; 4 Enterprise Key Laboratory of Anti-viral Drug Development, Guangdong, China; Chinese Academy of Medical Sciences & Peking Union Medical College, Beijing, China

**Keywords:** mass balance, pharmacokinetics, absorption, distribution, metabolism, metabolites, safety

## Abstract

Netanasvir Phosphate is a novel second-generation NS5A inhibitor used for the treatment of chronic hepatitis C virus (HCV) infection. This study investigated the absorption, distribution, metabolism, and excretion (ADME), and the metabolite profile of [¹⁴C]Netanasvir Phosphate. In a single-dose, open-label study, six healthy Chinese male volunteers received an oral dose of 100 mg/200 µCi of [¹⁴C]Netanasvir Phosphate under fed conditions. Blood, urine, and fecal samples were collected for up to 336 h post-dose. Total radioactivity was measured using oxidative combustion and liquid scintillation counting, and the parent drug and its metabolites were analyzed by validated liquid chromatography–tandem mass spectrometry and low-energy radiometric detection and mass spectrometry methods. The pharmacokinetic profile showed a median *T*_max_ of 5.5 h and a mean terminal half-life of 30.6 ± 8.52 h. The average total recovery of radioactivity was 97.73% ± 1.90%, with feces accounting for 97.71% of the administered dose and urine only 0.02%, indicating that fecal excretion is the primary elimination pathway. The parent drug was the predominant circulating and excreted component, representing 93.89% of plasma radioactivity and 78.78% of the administered dose in feces. Only minor metabolites were detected, including demethylated and oxidized derivatives. The drug was well tolerated, with no serious adverse events (SAEs) or dose-limiting toxicities observed. This mass balance and metabolite profiling study provides essential evidence supporting the favorable pharmacokinetic characteristics and metabolic stability of [¹⁴C]Netanasvir Phosphate and offers a scientific foundation for further clinical development in HCV-infected populations.

## INTRODUCTION

Hepatitis C virus (HCV) infection is a major global public health issue. According to an update released by the World Health Organization (WHO) in April 2016, the global chronic HCV infection rate is approximately 1.1%, with an estimated 100 million people affected worldwide (1). HCV is mainly transmitted through blood exposure, sexual contact, and vertical (mother-to-child) transmission. After acute infection, approximately 55%–85% of individuals will develop chronic hepatitis C (CHC) (2). Among the general population, 5%–15% of people may progress to liver cirrhosis within 20 years after infection, and the annual risk of hepatocellular carcinoma (HCC) in patients with cirrhosis is 2%–4% (3). HCV poses a serious threat to the health and survival of patients. The asymptomatic nature of CHC leads to low diagnosis and treatment rates, resulting in a large number of undetected infections and a sustained burden on the public health system (4, 5).

The emergence of direct-acting antiviral agents (DAAs) has fundamentally changed the treatment landscape for HCV infection, with cure rates exceeding 95% for various HCV genotypes (6). Nonstructural protein 5A (NS5A) is a key component of the HCV replication mechanism, playing a crucial role in viral RNA replication, viral particle assembly, and secretion. It also participated in various host cell processes, including interferon resistance and apoptosis regulation. Studies have reported that NS5A inhibitors can bind to the homodimers formed by NS5A at amino acid residues NS5A (aa 33–202) and/or NS5A (aa 25–215), thereby interfering with HCV replication (7). Netanasvir Phosphate is a second-generation NS5A inhibitor that has demonstrated potent pan-genotypic antiviral activity against HCV in a subgenomic replicon cell model. It is being developed for use in combination with other DAAs for the treatment of chronic hepatitis C in adult patients (aged 18 years and older).

The completed preclinical studies, including structural analysis, computer-aided molecular docking, *in vitro* drug resistance selection, and target specificity determination, indicated that the molecular target of Netanasvir Phosphate is the HCV NS5A protein. The compound exhibited potent pan-genotypic antiviral activity against HCV replicons. The cytotoxicity evaluations showed that the therapeutic index of Netanasvir Phosphate exceeded 4 × 10⁷ in human hepatocellular carcinoma cells, acute lymphoblastic leukemia cells, human embryonic lung fibroblasts, and renal epithelial cells, indicating a wide safety margin. In the completed phase I clinical trial, a single oral dose of 50–800 mg of Netanasvir Phosphate in healthy subjects was well tolerated, with no SAEs or dose-limiting toxicity observed. Under fed conditions, the time to reach the maximum plasma concentration (*T*_max_) was approximately 5.0–7.0 h. Within the tested dosage range, the drug exhibited either non-linear pharmacokinetics or unclear linearity. It also showed a long elimination half-life (*t*_1/2_), high plasma protein binding rate, and extensive tissue distribution, supporting its potential as a candidate for once-daily administration. The phase Ib trial in HCV-infected patients further confirmed its antiviral efficacy, with short-term monotherapy resulting in a significant reduction in HCV RNA levels. In addition, studies on drug interactions with Yiqibuvir have shown an increase in exposure to both drugs and/or their metabolites, but it is deemed unnecessary to adjust the dosage of combination therapy.

Although previous studies have laid a solid foundation for the clinical development of Netanasvir Phosphate, its absorption, distribution, metabolism, and excretion (ADME) characteristics in humans remain to be fully elucidated. Radiolabeled mass balance studies are essential for identifying the drug’s elimination pathways and major biotransformation routes. They facilitate comprehensive metabolite profiling, help determine whether disproportionate metabolites are present in human circulation, and assess the adequacy of preclinical pharmacological and toxicological evaluations (8). In addition, such studies can reveal potential metabolic interactions. Isotopic tracer techniques enable dynamic monitoring of drug distribution *in vivo*, providing insight into target engagement and off-target effects and serving as a valuable tool for early efficacy prediction (9). Radiolabeled and imaging-based tracer technologies play an irreplaceable role in mass balance studies, pharmacokinetic–pharmacodynamic evaluations, and clinical drug research. The present study was conducted in healthy Chinese male subjects to assess the mass balance after a single oral administration of [¹⁴C]Netanasvir Phosphate. The objectives were to characterize the drug’s absorption, excretion, and metabolic pathways and to evaluate its overall pharmacokinetic profile and safety, thereby providing a scientific basis for its further development.

## MATERIALS AND METHODS

### Study design

Netanasvir Phosphate (active pharmaceutical ingredient manufactured under GMP conditions) was supplied by Sunshine Lake Pharma Co., Ltd. (Batch No. 20221111-ZJ; Specification: 100 mg, expressed as Netanasvir Phosphate equivalents). [¹⁴C]Netanasvir Phosphate was provided by Shanghai WuXi AppTec Pharmaceutical Co., Ltd. The oral formulation of [¹⁴C]Netanasvir Phosphate consisted of a solid powder containing pharmaceutical excipients, sealed in glass vials, with each vial containing approximately 100 mg/200 µCi of [¹⁴C]Netanasvir Phosphate (expressed as Netanasvir Phosphate equivalents). The investigational product was stored at –10°C to –30°C in sealed, light-protected conditions following preparation.

This study was conducted in full compliance with the ethical principles outlined in the Declaration of Helsinki and the International Council for Harmonisation Good Clinical Practice (ICH-GCP) guidelines. The study protocol was reviewed and approved by the Institutional Ethics Committee of Shanghai Xuhui Central Hospital prior to implementation. Before conducting any study-related procedures, written informed consent has been obtained from all participants. The trial was registered with the Center for Drug Evaluation, National Medical Products Administration (https://www.chinadrugtrials.org.cn) under registration number CTR20222045.

Screening assessments were performed from Day −14 to Day −2 prior to dosing. Participants who met the preliminary eligibility criteria were admitted to the clinical research center two days before dosing. Upon admission, participants received training on study procedures—including drug administration and the collection of urine and fecal samples—to ensure compliance with the protocol and relevant standard operating procedures.

On the day prior to dosing, random pre-dose urine and fecal samples were collected as baseline specimens. On the morning of Study Day 1, baseline blood samples were collected within 1 h prior to dosing. The study drug ([¹⁴C]Netanasvir Phosphate, 100 mg/200 µCi) was administered orally 30 min after the start of a regular meal.

From 0 to 336 h post-dose, urine, feces, and blood samples were collected according to the protocol, and safety monitoring was conducted until the end of the study. A staged sampling approach was used, whereby the decision to terminate or extend biological sample collection was based on interim analytical results. Collection could be discontinued if the total radioactivity recovered from biological samples (urine + feces) exceeded 80% of the administered dose, and the amount recovered in two consecutive collection intervals was less than 1% of the dose. Plasma sample collection could be stopped if the blood radioactivity concentration fell below three times the baseline plasma value.

If the collection duration exceeded the initially planned period, sample collection would be extended at 24-h intervals (for urine and feces) or at 24-h multiples (e.g., 48 h, 72 h, for blood) until the predefined termination criteria were met. Safety monitoring continued until the final sample collection date.

Venous blood samples (~8 mL) were collected into EDTA-K₂ anticoagulant tubes at pre-dose (within 1 h before administration) and at 1.5, 3, 4, 5, 6, 7, 8, 12, 24, 48, 72, 96, 120, 168, 216, 264, and 336 h post-dose. After centrifugation, plasma was separated and primarily used for pharmacokinetic (PK) and metabolic profiling. Whole blood samples (~2 mL) were collected into EDTA-K₂ tubes at pre-dose and at 4, 6, 8, 12, 24, and 96 h post-dose for the determination of total radioactivity in whole blood. Additionally, extra venous blood samples (~10 mL) were collected at 4, 6, 8, 12, 24, and 96 h post-dose into EDTA-K₂ tubes. Plasma obtained after centrifugation was used for biotransformation analysis.

For all six participants, plasma PK sampling ended at 120 h post-dose. Urine collection was completed by 144 h in one participant and 72 h in the remaining five. Fecal samples were collected up to 240 h in four participants and 192 h and 216 h in the other two (one each), respectively. All sampling endpoints fulfilled the protocol-specified criteria for termination.

### Study participants

Healthy male volunteers aged 18–40 years with normal bowel habits were enrolled after providing written informed consent. Eligibility required the absence of clinically significant abnormalities, confirmed by physical examination, laboratory tests, ECG, and imaging. Participants were required to have a BMI between 19.0 and 28.0 kg/m² and agree to use effective contraception during the study and for 6 months thereafter. Subjects were excluded if they had any medical conditions or history that could affect drug absorption, distribution, metabolism, or excretion; recent use of medications or vaccines; participation in other clinical trials within the past 3 months; history of substance abuse, smoking, or alcohol dependence; or significant recent radiation exposure. Additional exclusion criteria included allergy to study-related substances, recent surgery, or any condition deemed unsuitable by the investigator.

### Pharmacokinetics

Plasma concentrations of Netanasvir Phosphate and its major metabolites at various time points were determined using a validated liquid chromatography–tandem mass spectrometry (LC-MS/MS) method. Pharmacokinetic parameters—including maximum plasma concentration (*C*_max_), *T*_max_, area under the plasma concentration–time curve from time zero to the last quantifiable time point (AUC_0–*t*_, obtained by the linear trapezoidal method), area under the curve extrapolated to infinity (AUC_0–∞_, AUC_0-∞_ = AUC_0–*t*_ + *C*_*t*_/λ_*z*_), terminal elimination half-life (*t*_1/2_, *t*_1/2 =_ ln (2)/*λ_z_*), apparent clearance (CL/F, CL/F = Dose/AUC_0–∞_), and apparent volume of distribution (V_z_/F, V_z_/F = Dose/(*λ_z_* × AUC_0–∞_))—were estimated using non-compartmental analysis with WinNonlin software (version 8.3, Pharsight). These parameters were summarized in tabular form and described using descriptive statistics.

### Excretion of radioactivity

Whole blood samples were thoroughly vortexed, and two parallel aliquots (~0.5 g each) were weighed into combustion boats. After drying, the samples were fully combusted using an oxidative combustion apparatus. The resulting [¹⁴C]CO₂ was captured in a ¹⁴C-specific scintillation solution, and radioactivity was quantified using a liquid scintillation counter. Plasma samples were vortexed, and a single aliquot (~0.5 g) was transferred to a 20 mL scintillation vial containing approximately 10 mL of scintillation solution. After thorough mixing, total radioactivity was measured using a liquid scintillation counter. Urine samples were well mixed, and a single aliquot (~2 g) was placed into a 20 mL scintillation vial with approximately 10 mL of scintillation solution and mixed uniformly. The total radioactivity was measured using a liquid scintillation counter. Fecal samples were transferred into pre-labeled containers of known tare weight. An appropriate volume of 50% aqueous isopropanol was added, and the samples were soaked for approximately 2–4 h or stored in a 4°C refrigerator (e.g., overnight). Prior to homogenization, additional isopropanol solution was added to achieve the desired consistency. The total weight was recorded, and samples were homogenized using a tissue homogenizer. Two parallel aliquots (~0.3 g each) of the homogenate were weighed into combustion boats, dried, and fully combusted using an oxidative combustion apparatus. The resulting [¹⁴C]CO₂ was captured in a ¹⁴C-specific scintillation solution and quantified using a liquid scintillation counter.

Whole blood and fecal samples were combusted using a biological oxidative combustion apparatus, and the resulting [¹⁴C]CO₂ was captured in 15 mL of ¹⁴C-specific scintillation solution and quantified using a liquid scintillation counter. Prior to sample combustion, a combustion recovery rate test was performed using a [¹⁴C]-labeled standard solution with a known radioactivity concentration to verify the efficiency of the oxidative combustion apparatus. The combustion recovery rate (>85.00%) was determined by comparing the radioactivity measured after complete combustion of the standard to that obtained by direct addition of the standard to the scintillation solution. Recovery rate assessments were conducted before, during, and after each day’s sample combustion. The daily mean recovery rate was calculated and archived in the instrument log. This average recovery rate was used as a correction factor: the radioactivity values (DPM) measured for all biological samples on that day were divided by the correction factor to obtain the actual radioactivity values. For each subject, the whole blood-to-plasma total radioactivity ratio (Kb/p) was calculated at each time point, and the mean and standard deviation of the ratio were determined across time points.

### Metabolite profiling and identification

#### Plasma

Based on the trapezoidal AUC (10) method and after excluding plasma samples with radioactivity below the lower limit of quantification, plasma from each time point of the six participants was pooled proportionally to generate six composite plasma samples for radioactive metabolite profiling. A portion of each pooled sample was extracted with acetonitrile (3:1, vol/vol), vortexed for 5 min, sonicated for 5 min, and stored at 4°C for 30 min. The mixture was then vortexed again for 2 min and centrifuged at 10,000 × *g* at 4°C for 10 min. The remaining pellet was first resuspended in 1  mL of ultrapure water by sonication, then extracted twice with methanol (2:1, vol/vol). The organic solvent extracts were combined, blown dry with nitrogen, and reconstituted with acetonitrile: ultrapure water (1:1). The reconstituted solution was centrifuged at 4°C and 10,000 × *g* for 10 min, and the supernatant was used for radio-metabolite profiling. Radioactivity in the organic extracts and reconstituted solutions was measured using a liquid scintillation counter. The post-extraction solid residue was dissolved by heating in 1 N KOH solution, followed by the addition of scintillation solution for radioactivity determination. All results were used to assess the recovery of radioactivity during the plasma extraction process.

#### Urine

Given the extremely low levels of radioactivity recovered in urine samples from all six participants (≤0.05% of the administered dose), with recoveries below 0.01% in subjects R02 and R05, urine samples collected during the 4–8 h, 8–12 h, and 12–24 h post-dose intervals were selected from the remaining four participants. Equal-volume aliquots were pooled within each subject based on time intervals to generate four composite urine samples: 4–12 h for subject R01, 4–24 h for subject R03, and 4–8 h for subjects R04 and R06. These samples were used for radioactive metabolite profiling. Portions of the pooled urine samples were lyophilized using a freeze-drying system and dissolved in ultrapure water. The mixture was vortexed at room temperature for 2 min, followed by extraction with acetonitrile (10×, vol/vol), vortexing for 5 min, and sonication for 5 min. Samples were then centrifuged at 10,000 × *g* for 10 min at 4°C. The resulting organic extracts were evaporated under nitrogen and reconstituted in methanol–water (1:9, vol/vol). After centrifugation at 10,000 × *g* and 4°C for 10 min, the supernatant was used for radio-metabolite profiling. Radioactivity in both the organic extracts and the reconstituted solutions was measured using a liquid scintillation counter. The solid residues remaining after extraction and reconstitution were dissolved by heating in 1 N KOH solution, and radioactivity was quantified following the addition of scintillation solution. All results were used to assess the recovery efficiency of radioactivity during the urine extraction process.

#### Feces

Calculations indicated that total radioactivity in fecal samples collected from 0 to 168 h accounted for 98.63% to 99.93% of the total radioactivity excreted over the full 0- to 240-h interval. Therefore, the 0- to 168-h fecal samples were considered representative of total fecal excretion. Fecal samples collected from each of the six participants during this period were pooled by time interval using equal weight proportions to generate six composite 0- to 168-h fecal homogenate samples for radioactive metabolite profiling. Part of the pooled fecal homogenates was extracted with acetonitrile (3×, vol/vol), vortexed at room temperature for 5 min, sonicated for 5 min, and centrifuged at 10,000 × *g* for 10 min at 4°C. The resulting residue was first resuspended in approximately 0.5 mL of ultrapure water by sonication, followed by methanol extraction (2×, vol/vol), repeated twice. The organic extracts were combined, blown dry using nitrogen, and then reconstituted with dimethyl sulfoxide:acetonitrile:ultrapure water (3:2:5). The reconstituted solution was centrifuged at 10,000 × *g* at 4°C for 10 min, and the supernatant was used for radio-metabolite profiling. Radioactivity in both the organic extracts and the reconstituted solutions was determined using a liquid scintillation counter. The post-extraction solid residues were combusted using an oxidative combustion apparatus, and the resulting [¹⁴C]CO₂ was captured in scintillation solution and measured. All data were used to evaluate the extraction efficiency and radioactivity recovery from fecal homogenate samples.

The overall recovery rates of radioactivity from plasma, urine, and feces using the described extraction procedures ranged from 87.61% to 105.88%, 95.45% to 109.83%, and 90.79% to 103.14%, respectively, indicating that radioactive components were efficiently extracted from all matrices. Major radioactive metabolites in pooled plasma, urine, and fecal samples were identified using high-performance liquid chromatography coupled with low-energy radiometric detection and mass spectrometry (LC-RAM/MS). Chromatographic separation was carried out on a Shimadzu CBM-20A HPLC system (Shimadzu, Japan), equipped with an ACE Excel 3 C18 analytical column (150 × 4.6 mm i.d., S/N: A271019). The mobile phase consisted of 0.4% aqueous formic acid, with pH adjusted to 3.20 using ammonia (mobile phase A), and acetonitrile (mobile phase B), delivered at a flow rate of 0.7 mL/min. The gradient elution program was as follows: 0–5 min, 0% B; increased to 25% B at 5 min; 50% B at 60 min; 100% B from 65 to 70 min; and returned to 0% B at 72 min for column re-equilibration. Extracted plasma, urine, and fecal samples were subjected to HPLC separation, and the HPLC streams were collected in 15-s intervals into Deepwell LumaPlate−96 plates using an automated fraction collector. Radioactivity in each fraction was determined using a microplate scintillation counter. Off-line radiometabolite profiles were obtained using ARCConvert and Evaluation software (version: ARC3 Ver 3.0.2.379). The radiochromatographic profile of each sample was subjected to integration to determine the peak area of each radioactive component. The relative proportion of each peak within the overall radioactivity profile was calculated and expressed as a percentage (%HPLC). Subsequently, the contribution of major metabolites to the total radioactivity in each corresponding biological matrix was derived. These key metabolites were further quantified as a percentage of total plasma radioactivity exposure (%AUC), calculated as: %AUC = (proportion of [¹⁴C]Netanasvir Phosphate and its metabolites in the plasma radiochromatogram) × (plasma extraction recovery of the sample). For urine and feces, the contribution of each metabolite was calculated as a percentage of the administered dose using the formula: %Dose = (total detected radioactivity in the sample [DPM]/total administered radioactivity [DPM]) × 100. Structural characterization of each radioactive metabolite was performed based on its radiochromatographic profile, retention behavior on HPLC, and known metabolic pathways. In addition, molecular weights and fragmentation patterns from mass spectrometry data were used to deduce the most probable structures of the detected radioactive metabolites.

### Safety and tolerability evaluation

Safety evaluations were conducted throughout the study and included the monitoring of treatment-emergent adverse events (TEAEs), clinical laboratory tests (hematology, biochemistry, urinalysis, and coagulation tests), vital signs (body temperature, blood pressure, heart rate, and respiratory rate), physical examination, and 12-lead electrocardiograms (ECGs). All adverse events (AEs) occurring after drug administration were recorded and assessed for severity and relationship to the study drug. TEAEs were coded using the Medical Dictionary for Regulatory Activities (MedDRA) and summarized by System Organ Class (SOC) and Preferred Term (PT).

### Statistical analysis

Pharmacokinetic (PK) parameters were calculated and statistically analyzed using WinNonlin software (version 8.3, Pharsight). Other data analyses were performed using SAS software (version 9.4, SAS Institute Inc., Cary, NC, USA). Descriptive statistics, including the number of subjects (n), mean, standard deviation (SD), coefficient of variation (CV%), median, interquartile range (IQR), minimum, maximum, geometric mean, and geometric coefficient of variation (gCV%), were used to summarize the total radioactivity concentrations in whole blood and plasma, as well as the concentrations of Netanasvir Phosphate at each blood sampling time point. Mean plasma and whole blood concentration–time profiles were plotted based on scheduled sampling times. All PK parameters were listed and summarized descriptively. The ratio of radioactivity concentrations between whole blood and plasma at each time point was calculated using Microsoft Excel (version 2010). For samples below the limit of quantification (BLQ), no ratio was calculated, and these values were excluded from the computation of mean ratios. Based on the actual administered radioactive dose, radioactivity concentrations, and the measured weight or volume of excreta of each subject, the percentage of radioactivity excretion, cumulative excretion, and total excretion during each time interval were calculated. Descriptive statistics, including mean and standard deviation (SD), were used to summarize the excretion rate data. A cumulative excretion curve was generated accordingly. For urine and fecal samples with radioactivity concentrations below the limit of quantification (BLQ), zero values were used when calculating excreted radioactivity. Categorical variables were summarized using frequency counts and percentages (*n*, %), such as for the tabulation of AEs.

## RESULTS

### Participants’ distribution and baseline characteristics

Six healthy male participants were enrolled in the study, and all successfully completed it. The mean (± SD) age and body mass index (BMI) were 28.5 ± 5.7 years and 24.6 ± 2.5 kg/m², respectively. Five participants were of Han ethnicity, and one was of Buyi ethnicity (Table 1).

**TABLE 1:** Demographics and baseline disease characteristics^*a*^

Parameter		Total (*N* = 6)
Age, years	Mean (SD)	28.5 (5.68)
	Median (range)	29.5 (25.0, 33.0)
Gender	Male	6 (100%)
Race	Asian	5 (83.3%)
	Others	1 (16.7%)
Height (cm)	Mean (SD)	169.25 (6.463)
Weight (kg)	Mean (SD)	70.62 (10.767)
BMI (kg/m^2^)	Mean (SD)	24.55 (2.497)

^*a*^ BMI, body mass index; SD, standard deviation.

### Pharmacokinetics

**Fig 1:**
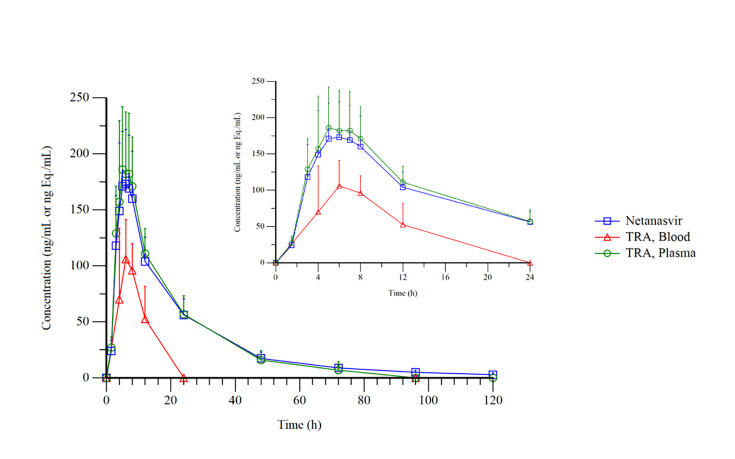
Mean concentration–time profiles (linear scale) of total radioactivity in whole blood and plasma, and parent Netanasvir in plasma after a single oral dose of 100 mg/200 µCi [¹⁴C]Netanasvir Phosphate in six healthy Chinese male subjects (mean + SD).

**Fig 2:**
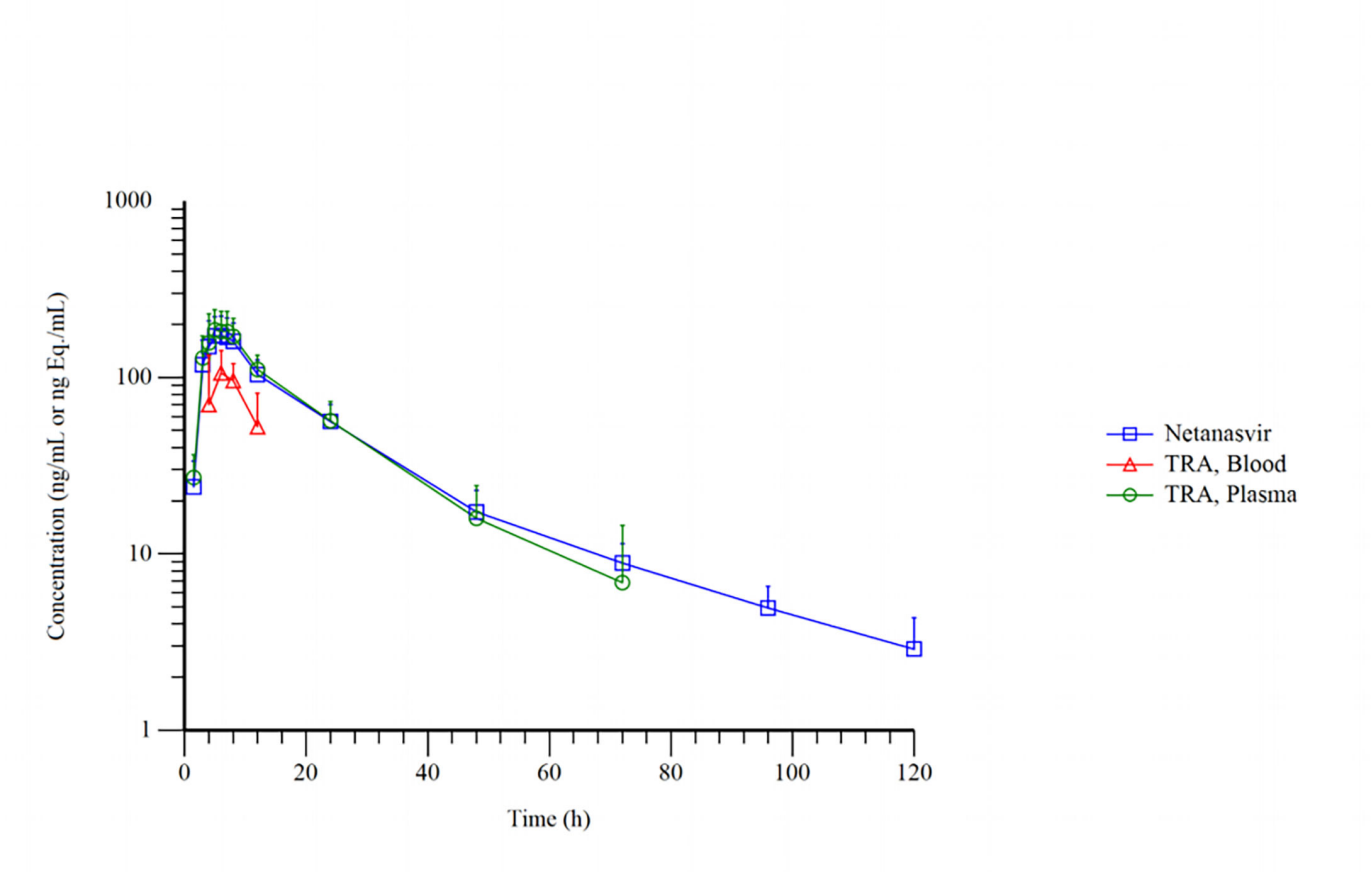
Mean concentration–time profiles (semi-logarithmic scale) of total radioactivity in whole blood and plasma, and parent Netanasvir in plasma after a single oral dose of 100 mg/200 µCi [¹⁴C]Netanasvir Phosphate in six healthy Chinese male subjects (mean + SD).

Following a single oral dose of approximately 100 mg/200 µCi [¹⁴C]Netanasvir Phosphate, the mean plasma and whole blood total radioactivity–time profiles, along with the concentration–time profile of the parent drug (Netanasvir Phosphate) in plasma, are presented in Fig. 1 (for linear coordinates) and Fig. 2 (for semi-logarithmic coordinates). After administration, the total radioactivity in plasma and whole blood, as well as the plasma concentration of the parent drug, rapidly reached peak levels and gradually decreased thereafter. The T_max_ was 6.00 (5.00–7.00) h, slightly later than that observed for the parent drug. The C_max_, expressed as Netanasvir Phosphate molar equivalents, was 193 ± 55.5 ng Eq./mL. The radioactivity concentrations subsequently declined, with quantifiable levels observed up to 72 h. The AUC_0–*t*_ was 3560 ± 1250 h·ng Eq./mL; mean residence time (MRT_₀–*t*_) was 16.6 ± 3.54 h; estimated mean terminal elimination half-life (*t*₁_/_₂) was 14.3 ± 2.74 h; the mean apparent volume of distribution (V_z_/F) was 547 ± 101 L; and the mean apparent clearance (CL/F) was 27.4 ± 8.30 L/h (Table 2).

**TABLE 2:** Pharmacokinetic parameters of total radioactivity and Netanasvir in plasma (means ± SD)^*a*^

Parameter	Unit	Total radioactivity	Netanasvir^*c*^
*T*_max_^*b*^	h	6.00 (5.00, 7.00)	5.50 (4.00, 7.00)
*C*_max_	ng eq./mL	193 ± 55.5	180,±,46.9
AUC_0*–t*_	h·ng eq./mL	3,560 ± 1,250	3,860 ± 974
AUC_0–∞_	h·ng eq./mL	3,930 ± 1,170	4,010 ± 970
MRT_0–*t*_	h	16.6 ± 3.54	23.8 ± 2.24
*t*_1/2_	h	14.3 ± 2.74	30.6 ± 8.52
V_z_/F	L	547 ± 101	1150 ± 419
CL/F	L/h	27.4 ± 8.30	26.0 ± 5.54

^*a*^ AUC_0–*t*_, area under the serum concentration–time curve from time 0 to the time of last quantifiable concentration; AUC_0–∞_, area under the serum concentration–time curve from time 0 extrapolated to infinity; CL, total clearance; *C*_max_, maximum observed serum concentration after administration; *T*_max_, time to maximum observed serum concentration after administration; *t*_1/2_, half-life; Vz/F, apparent volume of distribution. AUC_0–∞_ = AUC_0*–t*_ + *C*_last_/*λ*_*z*_.

^*b*^ *T*_max_ is presented as median (minimum, maximum).

^*c*^ The units of C_max_ , AUC_0*–t *_and AUC_0–∞_ for Netanasvir are ng/mL and ng·h/mL, respectively.

For the parent drug Netanasvir Phosphate in plasma, the *T*_max_ was 5.50 (4.00–7.00) h. The mean ± SD of *C*_max_ was 180 ± 46.9 ng/mL, followed by a gradual decline in plasma concentration, with quantifiable levels detected up to 120 h. The mean AUC_0–*t*_ was 3,860 ± 974 h·ng/mL; MRT_0–*t*_ was 23.8 ± 2.24 h; estimated mean *t*_₁/₂_ was 30.6 ± 8.52 h; mean V_z_/F was 1,150 ± 419 L; and mean CL/F was 26.0 ± 5.54 L/h (Table 2).

Total radioactivity in whole blood was assessed at time points from 0 to 96 h post-dose. Measurable radioactivity was detected only in samples collected between 4 and 12 h. The calculated whole blood-to-plasma total radioactivity ratios for quantifiable samples are summarized in Table 3. The mean ratios ranged from 0.539 to 0.580, indicating that [¹⁴C]Netanasvir Phosphate and its associated components exhibited minimal binding to blood cells.

**TABLE 3:** Cumulative excretion of radioactivity in urine and feces

Parameter	Subject						Total (mean ± SD)
	R01	R02	R03	R04	R05	R06	
Ae_urine_, % of dose	0.02	＜0.01^*a*^	0.05	0.01	＜0.01^*a*^	0.01	0.02 ± 0.02
Ae_feces_, % of dose	99.66	95.90	99.54	98.90	96.85	95.42	97.71 ± 1.89
Ae_total_, % of dose	99.68	95.90	99.59	98.91	96.85	95.43	97.73 ± 1.90

^*a*^ Values <0.01 were treated as 0.00 in calculations.

### Excretion of radioactivity in urine and feces

**Fig 3:**
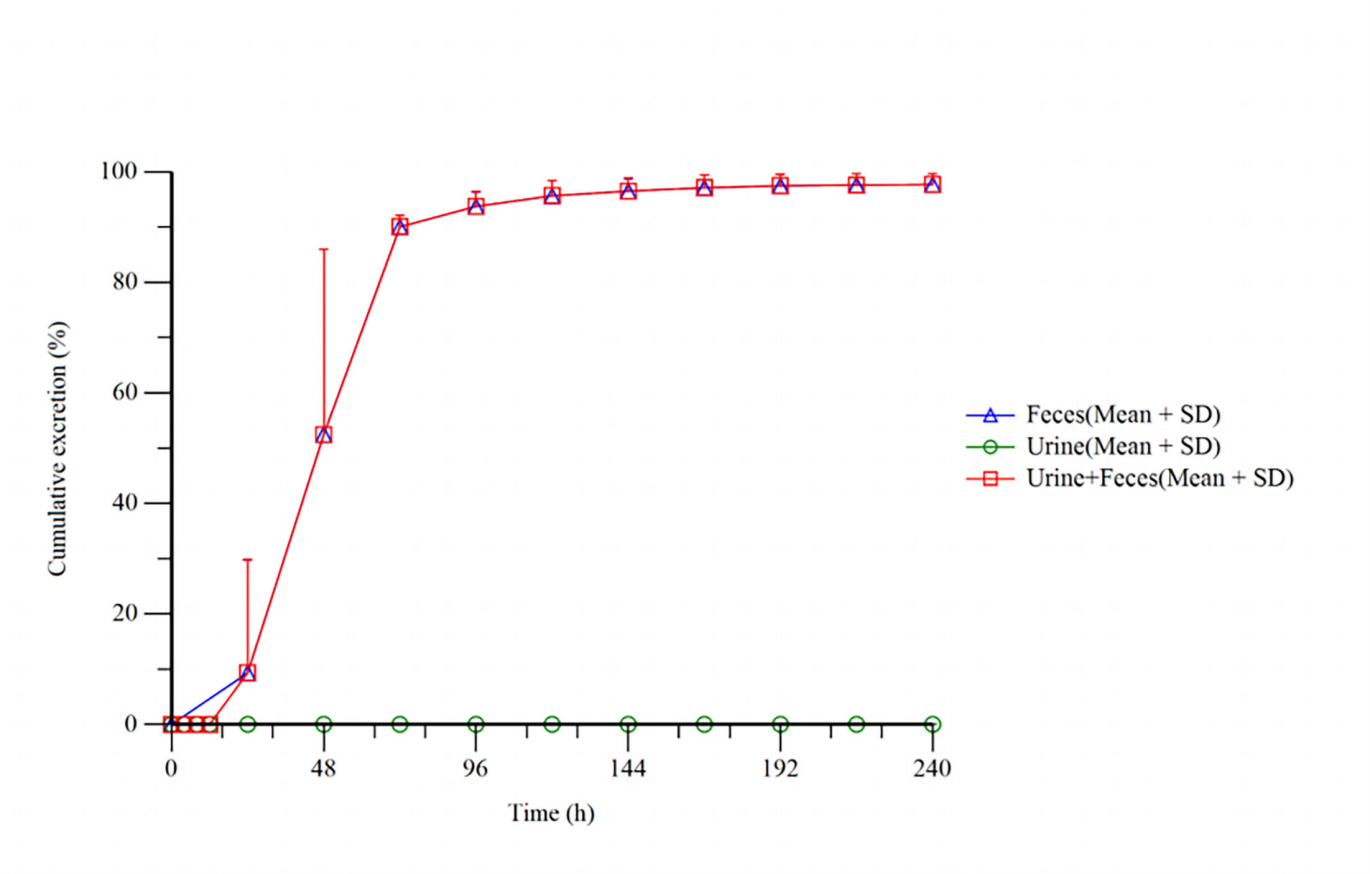
Cumulative excretion of radioactivity in urine and feces from subjects receiving a single oral dose of 100 mg/200 µCi [¹⁴C]Netanasvir Phosphate.

After a single oral administration of 100 mg/200 µCi [¹⁴C]Netanasvir Phosphate, the percentages of radioactivity recovered in the urine and feces of six healthy male participants are presented in Table 3, and the cumulative excretion profiles are shown in Fig. 3. The total radioactivity recovered from urine and feces accounted for 97.73% ± 1.90% of the administered dose, with fecal excretion accounting for the vast majority (97.71% ± 1.89%). The excretion in urine is very small, only 0.02% ± 0.02%. The excretion of total radioactivity mainly occurred within the first 72 h post-dose, during which more than 90.00% of the administered dose was eliminated. Beyond 120 h, the daily excretion fell below 1.00%.

### Metabolite profiling

**Fig 4:**
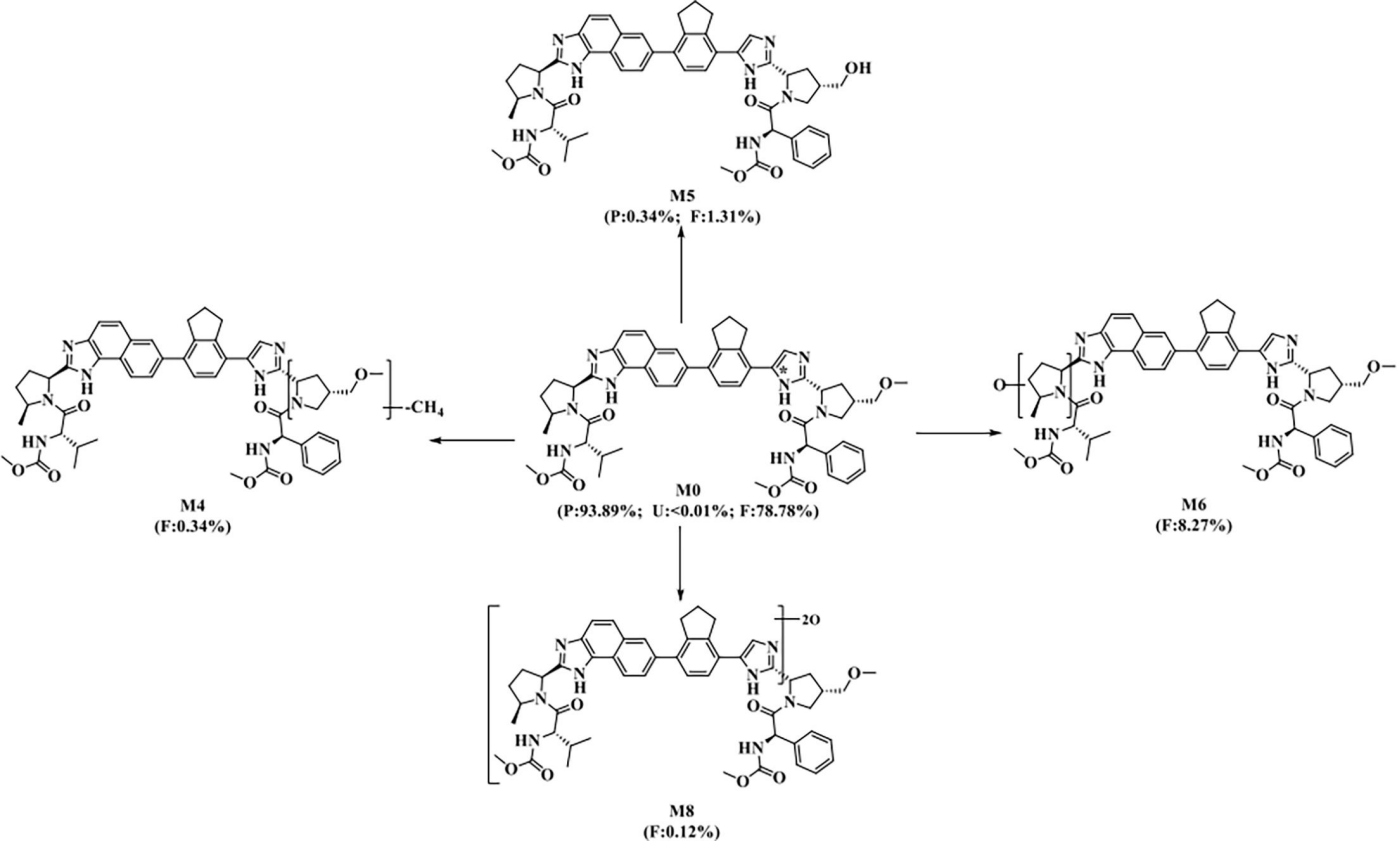
Proposed metabolic pathways of Netanasvir Phosphate in healthy male subjects.*: The position that 14C labelled,P: Plasma, expressed as %AUC, U: Urine, expressed as %Dose, F: Feces, expressed as %Dose.

Following a single oral administration of 100 mg/200 µCi [¹⁴C]Netanasvir Phosphate under fed conditions to six healthy Chinese adult male participants, metabolite profiling in human plasma, urine, and feces was conducted using an LC–MS/MS method. In addition to the parent drug, four metabolites were identified: a demethylated and dehydrogenated metabolite (M4), a demethylated metabolite (M5), a mono-oxidized metabolite (M6), and a di-oxidized metabolite (M8). These results suggest that [¹⁴C]Netanasvir Phosphate is primarily eliminated unchanged in humans, with oxidative and demethylation pathways contributing to its biotransformation. The proposed metabolic pathways are illustrated in Fig. 4. Metabolite profiling data for plasma and excreta are summarized in Table 4, and representative radiochromatograms of plasma and fecal samples are provided in Fig. S1.

**TABLE 4:** [^14^C] Netanasvir Phosphate and its main metabolites as percentage of total plasma radioactivity (%AUC) and percentage of dose in urine and feces (% dose)^*a*^^,^^*b*^

Metabolites	The type of metabolites	Plasma	Urine	Feces	Urine + feces
		%AUC	%Dose (0.02%^*c*^)	%Dose (97.71%^*c*^)	%Dose (97.73%^*c*^)
M0	Parent drug	93.89	<0.01	78.78	78.78
M4	Demethylated and dehydrogenated metabolites	ND	ND	0.34	0.34
M5	Demethylated metabolites	0.34	ND	1.31	1.31
M6	Monooxidative metabolites	ND	ND	8.27	8.27
M8	Di-oxidative metabolites	ND	ND	0.12	0.12
Total identified peaks^*d*^		94.23	NA	88.82	88.82
Total unidentified peaks^*e*^		5.77	NA	8.89	8.91
Percentage of peaks identified^*f*^		94.23	NA	90.90	90.88

^*a*^ %AUC = %HPLC of [¹⁴C]Netanasvir Phosphate and its metabolites in the plasma radiochromatogram × extraction recovery of the plasma sample.

^*b*^ ND: Not detected; NA: Not applicable.

^*c*^ Urinary excretion is expressed as the percentage of the administered dose recovered over 0–144 h, and fecal excretion over 0–240 h.

^*d*^ Percentage of identified peaks relative to total plasma radioactivity exposure (%AUC) or administered dose (%Dose).

^*e*^ Individual radioactive peaks account for no more than 1.50% of AUC or 5.00% of the administered dose.

^*f*^ The sum of identified peaks is expressed as the percentage of total radioactivity in the respective matrix.

Radioactive peaks identified in plasma accounted for 94.23% of the total circulating radioactivity. The parent drug was the predominant component in plasma, contributing 93.89% of total plasma radioactivity exposure, while the metabolite M5 accounted for only 0.34% (Table 4).

Total radioactivity excreted in urine over the 0- to 144-h interval represented just 0.02% of the administered dose. Due to the extremely low levels of radioactivity, only the parent compound was detected by mass spectrometry. Fecal excretion was the primary elimination pathway, with 97.71% of the administered dose recovered over 0–240 h. Identified radioactive peaks in feces represented 88.82% of the administered dose. The parent drug was the predominant component, accounting for 78.78% of the dose, while M6 was the major fecal metabolite, contributing 8.27%. The other minor metabolites—M4, M5, and M8—accounted for 0.34%, 1.31%, and 0.12% of the administered dose, respectively.

The peak at approximately 53.0 min in the HPLC chromatogram was identified as [¹⁴C]Netanasvir Phosphate. LC/(+)ESI-FTMS analysis of [¹⁴C]Netanasvir Phosphate produced a [¹²C-M+H]^+^ ion at m/z 895.4458, consistent with the molecular formula C₅₁H₅₉N₈O₇^+^ (mass error: –4.80 ppm). The major fragment ion spectrum derived from the [¹²C-M+H]^+^ ion is shown in Fig. S2. In the (+)HCD mode, the [M+H]^+^ ion also generated characteristic fragment ions as shown in Fig. S2. All observed fragment ions and the retention time were consistent with those of the [¹⁴C]Netanasvir Phosphate reference standard.

The peak at approximately 48.5 min in the HPLC chromatogram was identified as M4. LC/(+)ESI-FTMS analysis of M4 produced a [¹²C-M+H]^+^ ion at m/z 879.4155, which is consistent with the molecular formula C₅₀H₅₅N₈O₇^+^ (mass error: –3.75 ppm). The major fragment ion spectrum generated from the [¹²C-M+H]^+^ ion is shown in Fig. S3. In the (+)HCD mode, cleavage of C–C and C–N bonds in M4 yielded a fragment ion at m/z 98, while C–N bond cleavage combined with N-demethylation generated a fragment ion at m/z 671; further C–C and C–N bond cleavage of the m/z 671 ion produced a fragment ion at m/z 417. The formation of fragment ions at m/z 130 and m/z 164 was consistent with that observed for the [¹⁴C]Netanasvir Phosphate reference standard. The proposed MS/MS fragmentation pathway of M4 is shown in Fig. S3, and all fragment ions were consistent with the structure of M4.

The peak at approximately 43.0 min in the HPLC chromatogram was identified as M5. LC/(+)ESI-FTMS analysis of M5 produced a [¹²C-M+H]^+^ ion at m/z 881.4308, consistent with the molecular formula C₅₀H₅₇N₈O₇^+^ (mass error: –4.20 ppm). The major fragment ion spectrum generated from the [¹²C-M+H]^+^ ion is shown in Fig. S4. In the (+)HCD mode, the fragmentation pattern of M5 was generally consistent with that of the [¹⁴C]Netanasvir Phosphate reference standard. Specifically, fragment ions at m/z 100 and m/z 673 were identified as demethylated forms of fragment ions at m/z 114 and m/z 687, respectively. The proposed fragmentation pathway of M5 is illustrated in Fig. S4, and all observed fragment ions were consistent with the structure of M5.

The peak at approximately 40.0 min in the HPLC chromatogram was identified as M6. LC/(+)ESI-FTMS analysis of M6 produced a [¹²C-M+H]^+^ ion at m/z 911.4411, consistent with the molecular formula C₅₁H₅₉N₈O₈^+^ (mass error: –4.28 ppm). The major fragment ion spectrum generated from the [¹²C-M+H]^+^ ion is shown in Fig. S5. In the (+)HCD mode, the fragmentation pattern of M6 was generally consistent with that of the [¹⁴C]Netanasvir Phosphate reference standard. Fragment ions at m/z 433, 607, 671, 703, and 879 were identified as mono-oxidized counterparts of fragment ions at m/z 417, 591, 655, 687, and 863, respectively. Notably, the fragment ion at m/z 433 could also undergo dehydration to form a fragment ion at m/z 415. The proposed fragmentation pathway of M6 is illustrated in Fig. S5, and all fragment ions were consistent with the structure of M6.

The peak at approximately 36.5 min in the HPLC chromatogram was identified as M8. LC/(+)ESI-FTMS analysis of M8 produced a [¹²C-M+H]^+^ ion at m/z 927.4377, consistent with the molecular formula C₅₁H₅₉N₈O₉^+^ (mass error: –2.48 ppm). The major fragment ion spectrum generated from the [¹²C-M+H]^+^ ion is shown in Fig. S6. In the (+)HCD mode, the fragmentation pattern of M8 was generally consistent with that of the [¹⁴C]Netanasvir Phosphate reference standard. Among these, fragment ions at m/z 623 and m/z 687 were identified as di-oxidized forms of fragment ions at m/z 591 and m/z 655, respectively. The proposed fragmentation pathway of M8 is shown in Fig. S6, and all fragment ions were consistent with the structure of M8.

### Safety and tolerability

A total of five participants (83.3%, 5/6) experienced six TEAEs, among which five TEAEs reported in four participants (66.7%, 4/6) were deemed related to the study drug. The severity of all TEAEs was Grade 1; no TEAEs ≥grade 2 level were observed. Except for one event, which was managed with non-pharmacological intervention (cooling therapy for COVID-19 infection), no treatment was required. All TEAEs resolved completely without any sequelae. No SAEs, discontinuations due to AEs, or deaths occurred during the study.

## DISCUSSION

This study provides the first comprehensive assessment of the pharmacokinetics, metabolism, and excretion of [^14^C]Netanasvir Phosphate, a novel second-generation NS5A inhibitor, in healthy male subjects. Following a single oral dose, the drug exhibited favorable pharmacokinetic properties, including rapid absorption (median *T*_max_ of 5–7 h), high plasma protein binding, and a prolonged terminal half-life, which supports a once-daily dosing regimen. The parent compound was the predominant circulating moiety, and fecal elimination was the primary route of excretion, accounting for approximately 97.71% of the administered dose, with negligible renal excretion (<0.05%). These findings indicate that Netanasvir Phosphate undergoes minimal renal clearance and that its systemic elimination occurs predominantly via hepatobiliary pathways.

Metabolite profiling identified four minor metabolites (M4, M5, M6, and M8), formed mainly via oxidative and demethylation pathways. These metabolic routes are consistent with those observed in other NS5A inhibitors, such as ombitasvir, which are known to undergo minimal CYP-mediated biotransformation (11, 12). The low abundance of metabolites relative to the parent drug in both plasma and feces suggests limited metabolic transformation. The predominance of unchanged drug in systemic circulation and excreta is indicative of high metabolic stability and reduced likelihood of disproportionate metabolites, which are important considerations in drug safety evaluations and regulatory review (13). Furthermore, the minor role of renal excretion suggests that dosage adjustments may not be necessary in patients with renal impairment, although this requires further investigation.

When compared with other approved NS5A inhibitors such as ledipasvir, velpatasvir, and ombitasvir, the pharmacokinetic and excretion characteristics of Netanasvir Phosphate appear class-consistent. For example, ledipasvir demonstrates minimal renal clearance and is primarily eliminated unchanged via feces, with a *t*_1/2_ of 47 h (14). Velpatasvir and ombitasvir also exhibit high plasma protein binding and biliary excretion with limited metabolism (11, 15). Notably, in contrast to ledipasvir, which produces several circulating oxidative and glucuronidated metabolites, Netanasvir phosphate showed a simpler metabolic profile, which could potentially translate into a lower risk of metabolic drug–drug interactions (DDIs).

This elimination pattern—characterized by predominant fecal excretion of the parent compound with negligible renal contribution—is largely attributed to the high lipophilicity and extensive plasma protein binding of NS5A inhibitors, such as ledipasvir and velpatasvir, whose protein binding exceeds 99%. These physicochemical properties limit renal filtration and favor hepatic uptake, followed by biliary excretion mediated by canalicular efflux transporters, notably P-glycoprotein (P-gp) and breast cancer resistance protein (BCRP) (14, 15). From both safety and efficacy perspectives, predominant fecal elimination of the unchanged drug may offer several advantages. First, clearance of the parent compound with minimal biotransformation reduces metabolic burden, including the formation of potentially reactive intermediates, which may lower the risk of metabolite-related toxicity (11, 13). Second, stable systemic exposure, as observed for Netanasvir Phosphate in the present study and for other approved NS5A inhibitors, supports sustained antiviral activity with predictable pharmacokinetics (14–16). In addition, reliance on biliary rather than renal elimination reduces dependency on renal function for drug clearance, which may be advantageous in patients with renal impairment. Nevertheless, hepatic dysfunction may alter biliary excretion and systemic exposure, warranting further pharmacokinetic evaluation in populations with compromised liver function (11, 13, 14).

A previously completed drug–drug interaction study involving Netanasvir Phosphate and the NS5B inhibitor Encofosbuvir (a sofosbuvir analog) demonstrated that coadministration led to modest increases in systemic exposure of both drugs and their metabolites. These changes were not considered clinically significant and did not necessitate dose adjustments, consistent with similar findings for ledipasvir–sofosbuvir and velpatasvir–sofosbuvir fixed-dose combinations (16–18). This supports the use of Netanasvir Phosphate in potential combination regimens without the need for additional pharmacokinetic optimization.

The current study design incorporated gold-standard methodology for human ADME studies, including radiolabeled drug administration, serial collection of excreta, and validated LC-RAM/MS techniques to identify and quantify parent drug and metabolites. The high total recovery of radioactivity (>97%) and the resolution of individual metabolic species enhance the robustness of the findings. Nevertheless, the study has some limitations. First, the inclusion of only healthy male volunteers limits generalizability to broader patient populations, particularly women, elderly patients, and those with hepatic or renal impairment. Second, the study did not assess the pharmacologic activity or toxicity of the minor metabolites, although their low abundance reduces the likelihood of clinical relevance.

Future research should be extended to include patients with chronic HCV infection and special populations, such as individuals with hepatic or renal impairment, elderly patients, and females. It is also recommended to further investigate the pharmacological activity and toxicological profiles of the identified metabolites to clarify their potential contributions to the overall safety of the drug. In addition, given that certain NS5A inhibitors have demonstrated altered pharmacokinetic behavior in patients with hepatic cirrhosis, dedicated studies in this population will be essential to enable precision dosing and ensure safe and effective use (19, 20).

### Conclusion

In conclusion, this first-in-human mass balance study of [^14^C]Netanasvir Phosphate indicates that the drug is rapidly absorbed, minimally metabolized, and primarily eliminated via the fecal route as an unchanged parent compound. The favorable pharmacokinetic characteristics—such as high metabolic stability, negligible renal clearance, and predominant biliary excretion—support once-daily oral administration and indicate a lower likelihood of drug–drug interactions or accumulation of toxic metabolites. The identification of only minor oxidative and demethylated metabolites further indicates a simple and predictable metabolic profile. These findings, combined with prior single- and multiple-dose clinical data, establish a strong pharmacological foundation for continued development of Netanasvir Phosphate as a potent and safe next-generation NS5A inhibitor. Further studies in HCV-infected patients and special populations, as well as additional pharmacodynamic and safety evaluations of its metabolites, will be critical for confirming its clinical potential and guiding its future therapeutic application.

## SUPPLEMENTAL MATERIAL

Fig. S1 aac.01655-25-s0001.pdf Representative radio-chromatograms of the metabolites.

Fig. S2 aac.01655-25-s0002.pdf Full-Scan MS/MS spectrum of netanasvir phosphate.

Fig. S3 aac.01655-25-s0003.pdf Full-Scan MS/MS spectrum and proposed fragmentation pathways of metabolite M4.

Fig. S4 aac.01655-25-s0004.pdf Full-Scan MS/MS spectrum and proposed fragmentation pathways of metabolite M5.

Fig. S5 aac.01655-25-s0005.pdf Full-Scan MS/MS spectrum and proposed fragmentation pathways of metabolite M6.

Fig. S6 aac.01655-25-s0006.pdf Full-Scan MS/MS spectrum and proposed fragmentation pathways of metabolite M8.

ASM does not own the copyrights to Supplemental Material that may be linked to, or accessed through, an article. The authors have granted ASM a non-exclusive, world-wide license to publish the Supplemental Material files. Please contact the corresponding author directly for reuse.
