# Supplementary material for: Mass balance study of [14C]Netanasvir Phosphate in healthy Chinese participants
Source: Antimicrob Agents Chemother. 2026 Apr 20;70(6):e01655-25. doi: 10.1128/aac.01655-25 (PMC13231878; doi:10.1128/aac.01655-25)
Supplement: Fig. S1 — Representative radio-chromatograms of the metabolites. [file aac.01655-25-s0001.pdf]

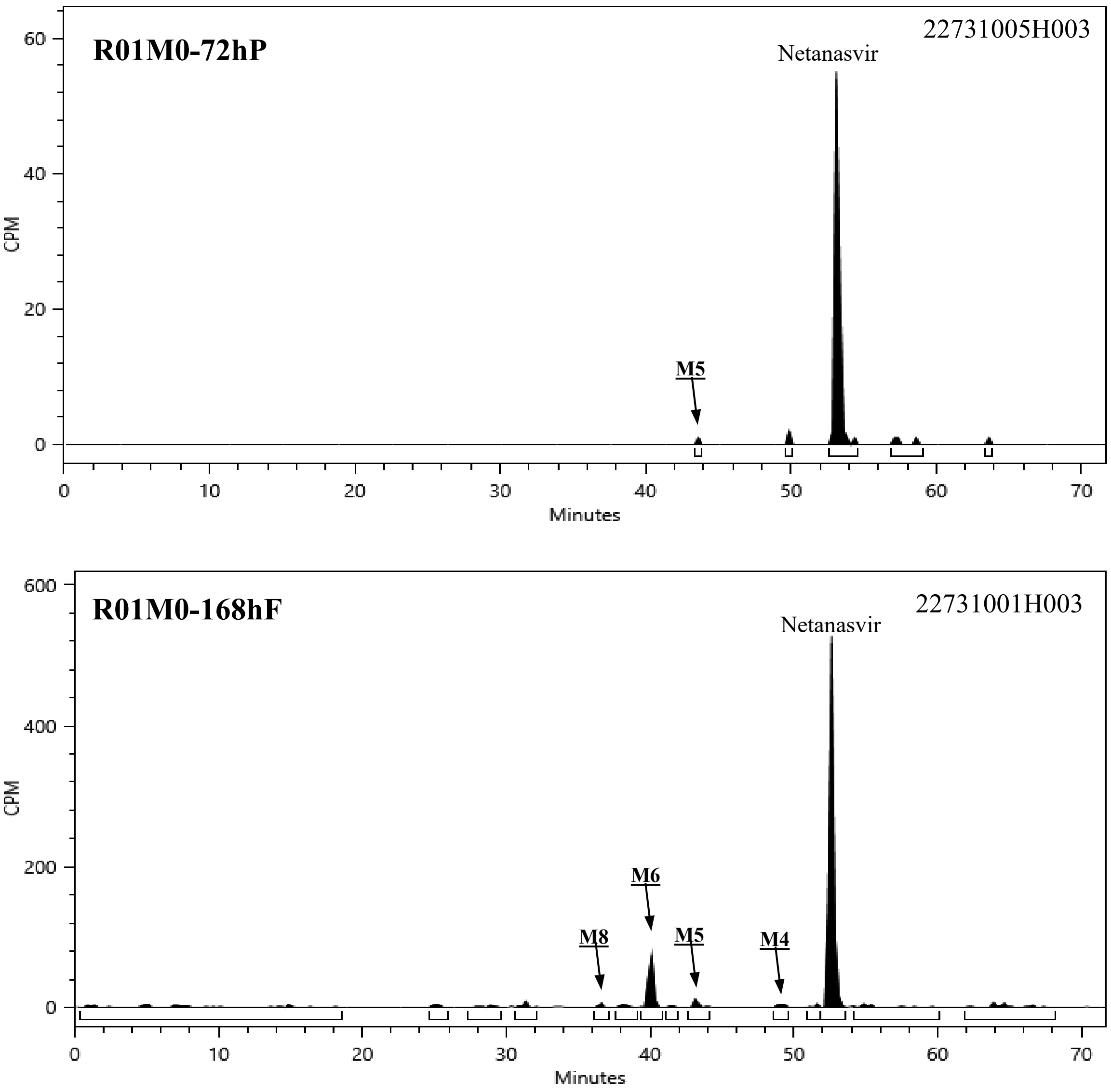

Supplementary Figure 1. Representative radio-chromatograms of the metabolites in human plasma and fecal samples
