# Supplementary figures and images for: Mass balance study of [14C]Netanasvir Phosphate in healthy Chinese participants
Source: Antimicrob Agents Chemother. 2026 Apr 20;70(6):e01655-25. doi: 10.1128/aac.01655-25 (PMC13231878; doi:10.1128/aac.01655-25)

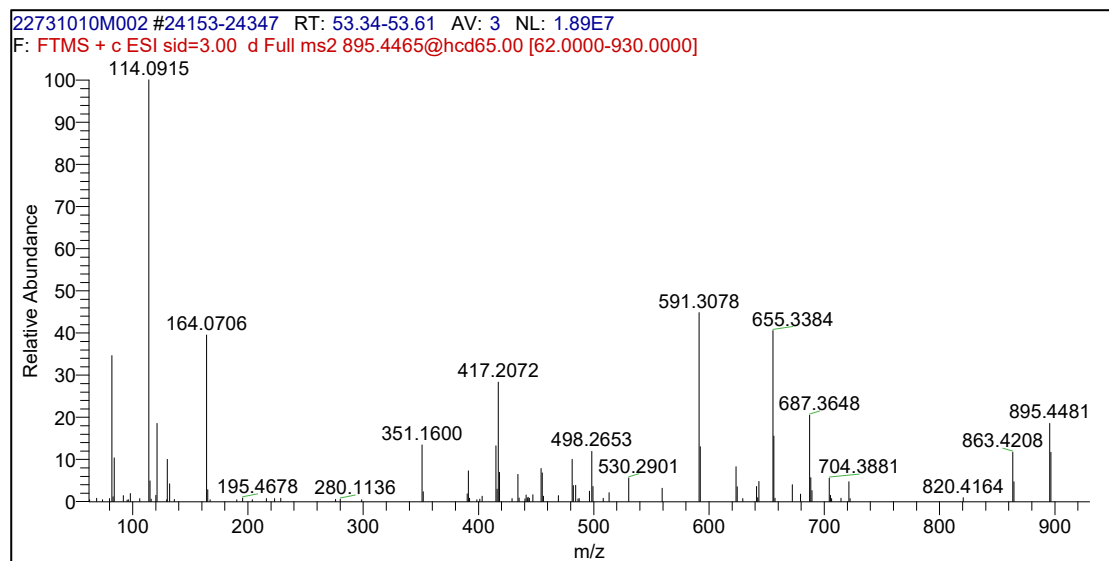

Supplementary Figure 2. (+)HCD-FTMS Full-Scan MS/MS Spectrum of Netanasvir Phosphate

Supplement: Fig. S2 — Full-Scan MS/MS spectrum of netanasvir phosphate. [file aac.01655-25-s0002.pdf]
