# Supplementary material for: Mass balance study of [14C]Netanasvir Phosphate in healthy Chinese participants
Source: Antimicrob Agents Chemother. 2026 Apr 20;70(6):e01655-25. doi: 10.1128/aac.01655-25 (PMC13231878; doi:10.1128/aac.01655-25)
Supplement: Fig. S4 — Full-Scan MS/MS spectrum and proposed fragmentation pathways of metabolite M5. [file aac.01655-25-s0004.pdf]

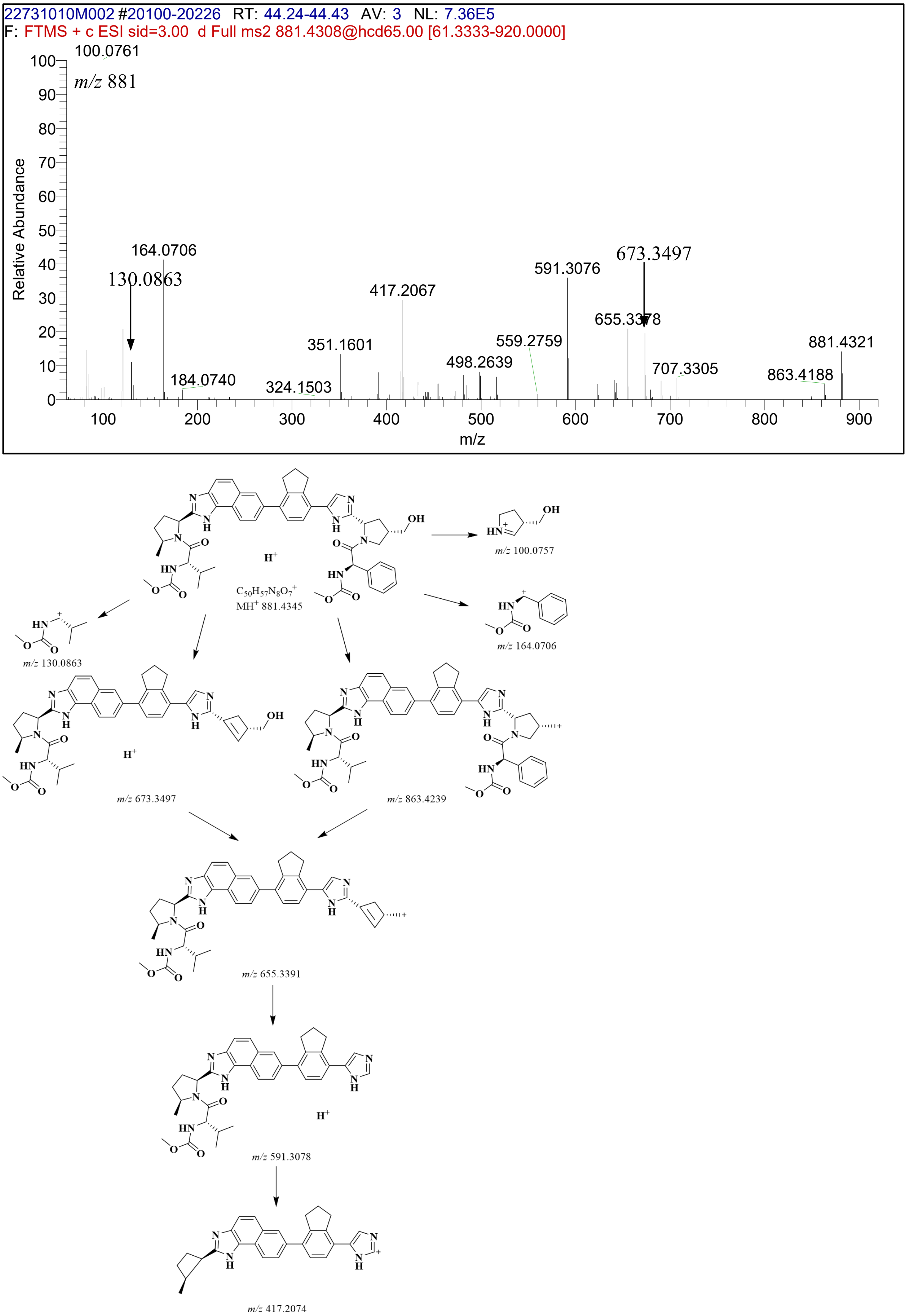

Supplementary Figure 4. (+)HCD-FTMS Full-Scan MS/MS Spectrum and Proposed Fragmentation Pathways of Metabolite M5
