# Supplementary material for: Mass balance study of [14C]Netanasvir Phosphate in healthy Chinese participants
Source: Antimicrob Agents Chemother. 2026 Apr 20;70(6):e01655-25. doi: 10.1128/aac.01655-25 (PMC13231878; doi:10.1128/aac.01655-25)
Supplement: Fig. S5 — Full-Scan MS/MS spectrum and proposed fragmentation pathways of metabolite M6. [file aac.01655-25-s0005.pdf]

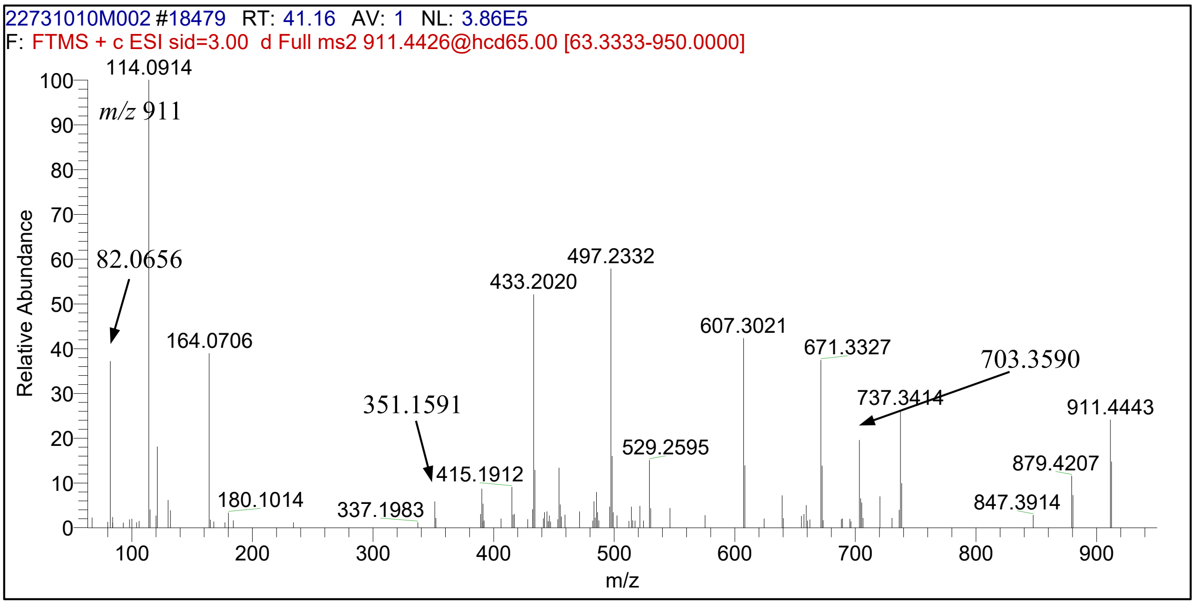

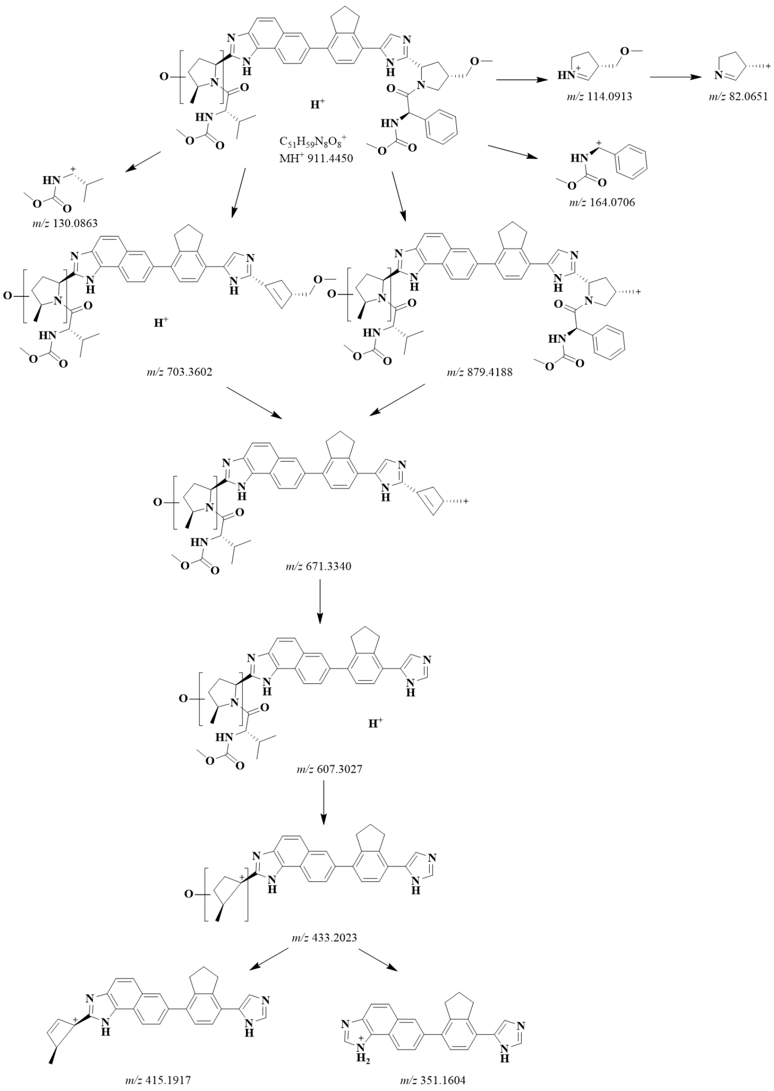

Supplementary Figure 5. (+)HCD-FTMS Full-Scan MS/MS Spectrum and Proposed Fragmentation Pathways of Metabolite M6
